# Effects of Apatinib on the “Stemness” of Non-Small-Cell Lung Cancer Cells In Vivo and Its Related Mechanisms

**DOI:** 10.1155/2020/2479369

**Published:** 2020-08-10

**Authors:** Bin Yang, Yan Wang, Zhuoying Chen, Yi-Ming Feng, Liang-Liang Shi

**Affiliations:** ^1^Department of Oncology, Hubei Cancer Hospital, Tongji Medical College, Huazhong University of Science and Technology, Wuhan 430079, China; ^2^Department of Oncology, Liyuan Hospital, Tongji Medical College, Huazhong University of Science and Technology, Wuhan 430077, China; ^3^Department of Geriatrics, Liyuan Hospital, Tongji Medical College, Huazhong University of Science and Technology, Wuhan 430077, China; ^4^Department of Radiology, Union Hospital, Tongji Medical College, Huazhong University of Science and Technology, Wuhan 430022, China; ^5^Cancer Center, Union Hospital, Tongji Medical College, Huazhong University of Science and Technology, Wuhan 430022, China

## Abstract

**Objective:**

To investigate the effects of Apatinib on the “stemness” of lung cancer cells in vivo and to explore its related mechanisms.

**Methods:**

A xenograft model of lung cancer cells A549 was established in nude mice and randomized into a control group (*n* = 4) and an Apatinib group (*n* = 4). Tumor tissues were harvested after 2 weeks, and mRNA was extracted to detect changes in stemness-related genes (*CD133*, *EPCAM*, *CD13*, *CD90*, *ALDH1*, *CD44*, *CD45*, *SOX2*, *NANOG*, and *OCT4*) and Wnt/*β*-catenin, Hedgehog, and Hippo signal pathways.

**Results:**

Compared with the control group, the volume and weight of nude mice treated with Apatinib were different and had statistical significance. Apatinib inhibited the expressions of *ABCG2*, *CD24*, *ICAM-1*, *OCT4*, and *SOX2* and upregulated the expressions of *CD44*, *CD13*, and *FOXD3*. Apatinib treatment also inhibited the Wnt/*β*-catenin, Hedgehog, and Hippo signaling pathways.

**Conclusion:**

Apatinib suppressed the growth of non-small-cell lung cancer cells by repressing the stemness of lung cancer through the inhibition of the Hedgehog, Hippo, and Wnt signaling pathways.

## 1. Introduction

Lung cancer is one of the most common malignant tumors in the world. Approximately 1.8 million people are diagnosed with lung cancer every year, with 1.6 million people dying from the deadly disease [1]. The 5-year survival rate for lung cancer is still less than 20%, although considerable progress has been made in various treatments, such as surgery, radiotherapy, chemotherapy, and targeted therapy. Targeted therapy is one of the essential treatment methods for advanced non-small-cell lung cancer [2]. At present, epidermal growth factor receptor (*EGFR*) tyrosine kinase inhibitors (*TKI*), anaplastic lymphoma kinase (*ALK*) tyrosine kinase inhibitors and *ROS1* inhibitors, epidermal growth factor receptor (*EGFR*) inhibitors, mesenchymal-epithelial transition factor (*MET*) inhibitors, epidermal growth factor receptor 2 (*EGFR*) inhibitors, murine sarcoma virus oncogene congener B1 (*B1BRAF*) inhibitors, Kristen murine sarcoma virus oncogene homologues (*KRAS*) inhibitors, and other targeted drugs are emerging. Targeted drugs have significantly improved and prolonged the overall survival (OS) and progression-free survival (PFS) of patients [3]. However, each targeted drug is only suitable for lung cancer patients with specific genetic mutations, and since many patients do not have the above known genetic mutations, they cannot receive targeted therapy and, thus, have a short survival time.

Apatinib is an antiangiogenic drug developed independently in China. Apatinib has been used before for the second-line treatment of gastric cancer. Studies have shown that Apatinib can also be used in the treatment of liver cancer after chemotherapy has failed [4]. The main antitumor mechanism of the drug is to inhibit tumor growth by inhibiting vascular endothelial growth factor 2 (*VEGFR*-*2*) and then inhibiting tumor angiogenesis [5]. Previous investigations have indicated that antiangiogenic drugs can inhibit the growth of multiple types of solid tumors, including non-small-cell lung cancer (NSCLC) [6]. Wang et al. included 128 patients with NSCLC with the failure of first-line treatment. Their results showed that patients who received a combined therapy of Apatinib and chemotherapy had significantly prolonged PFS and OS compared to those who underwent chemotherapy alone [7].

Lung cancer stem-like cells belong to a subpopulation of undifferentiated lung tumor cells that possess the ability to proliferate indefinitely and renew themselves, which is critical for lung cancer tumorigenesis, metastasis, resistance to therapy, and disease relapse [8]. Some studies have shown that lung cancer stem cells are the origin of malignant manifestations of lung cancer. Lung cancer stem cells can promote tumor proliferation through Hedgehog, Wnt, Hippo, and other signaling pathways. Moreover, lung cancer stem cells express “stemness”-related biomarkers, such as *CD44*, *CD24*, *CA133*, *CD13*, *CD90*, *ABCG2*, *SOX2*, and *EPCAM* [9]. Therefore, new therapeutic strategies targeting these signaling pathways could contribute to the regulation of stem cell replication, survival, and differentiation.

In this study, we established a subcutaneous NSCLC xenotransplantation in nude mice to observe the effects of Apatinib on the expression of surface markers of lung cancer stem cells, totipotent factors, and stemness-related genes and signal pathways, providing theoretical bases for drug selection of advanced non-small-cell lung cancer.

## 2. Materials and Methods

### 2.1. Animals and NSCLC Xenotransplantation

We purchased four-to-five-week-old female and male BALB/c nude mice from Vital River Laboratory Animal Technology Co. Ltd. (Beijing, China). All animal experiments were carried out in line with the National Institutes of Health guide for the care and use of laboratory animals. Approximately 1 × 10^7^ A549 cells were suspended in 1 ml phosphate-buffered solution (PBS), and the cell suspension (0.2 mL) was injected subcutaneously into the right axillary of nude mice and then reared in a cage under SPF condition.

After the inoculation of all nude mice, the general condition and tumorigenesis of the inoculation sites were observed and recorded every day. Eight nude mice (7 days after tumor inoculation) were randomized into a control group (*n* = 4) and an Apatinib group (*n* = 4). The two groups of mice were subjected to treatment by oral gavage once daily for 14 consecutive days, as follows: (a) Apatinib (50 mg/kg) (Hengrui Pharmaceutical company, Jiangsu, China); (b) DMSO (100 *μL*/20 g) (MP Biomedicals, USA); the maximum diameter (A) and the minimum diameter (B) of the tumors were measured with calipers on the 1^st^, 5^th^, 8^th^, 11^th^, and 14^th^ day after the drug administration and 24 hours after the end of the gavage, and the tumor volume, (*V*)=(*A* × *B*^2^)/2, was calculated. The tumor tissues from the A549 xenografts were separated and collected after the last drug administration on day 14. These tumor tissues were frozen immediately in liquid nitrogen and stored for qPCR analysis. The growth curves of the xenotransplantation tumors of the experimental and control groups were plotted using GraphPad Prism 7.

### 2.2. Detecting the Expression Level of “Stemness”-Related Genes in Tumor Tissues by Real-Time PCR

100 mg of subcutaneous xenograft tissues from each of the Apatinib group and the control group was used for this investigation. The total RNA was extracted using RNA extraction reagent Trizol (Life Technologies, Grand Island, NY, USA) following the manufacturer's instructions. Reverse transcription was performed with PrimeScript RT Reagent Kit (Vazyme, Thermo Fisher, NY, USA). qRT-PCR was carried out with the SYBR® Green PCR Master Mix (Life Technologies, Grand Island, NY, USA) in conformity with the manufacturer's instructions. The sequences for sense (S) and antisense (A) primers are shown in Table 1.

### 2.3. Statistical Analysis

The SPSS 22.0 statistical software (SPSS Inc., Chicago, IL, USA) was used for the analysis of statistical data. Data are expressed as mean ± standard deviation (*x* ± *s*). Two-group comparisons were made using the independent-samples *t*-test, while multiple group comparisons were conducted using the least significant difference test. Values of *P* < 0.05 were considered statistically significant.

## 3. Results

### 3.1. Comparison of Growth of Subcutaneous Xenografts between Apatinib and Control Groups

**Figure 1:**
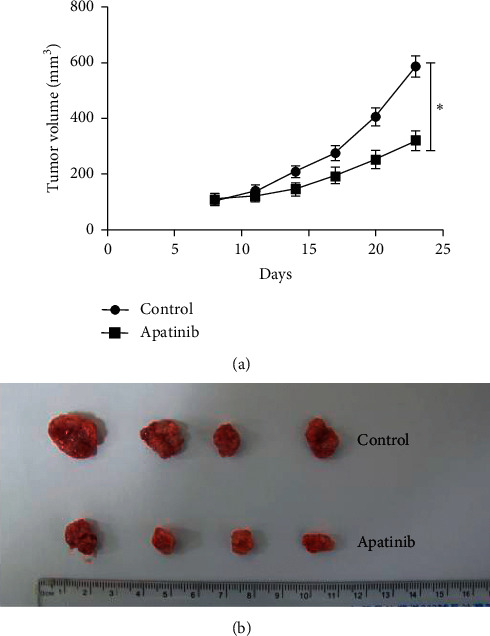
Effect of Apatinib on the volume change of subcutaneous transplanted tumor in nude mice, ^*∗*^*P* < 0.05.

Drugs were administered when the tumor volume of the nude mice in the two groups reached about 100 mm^3^. The body weight and tumor volume were measured and recorded on days 8, 11, 14, 17, and 20 after inoculation; the volume change curve is shown in Figure 1(a). The results indicated that the growth and volume of tumors were slower and smaller, respectively, in the Apatinib group than in the control group (*P* < 0.05). The final tumor masses of the two groups were compared as shown below (Figure 1(b)).

### 3.2. Expression of Stemness-Related Genes in NSCLC Subcutaneous Xenografts in Nude Mice at mRNA Level

**Figure 2:**
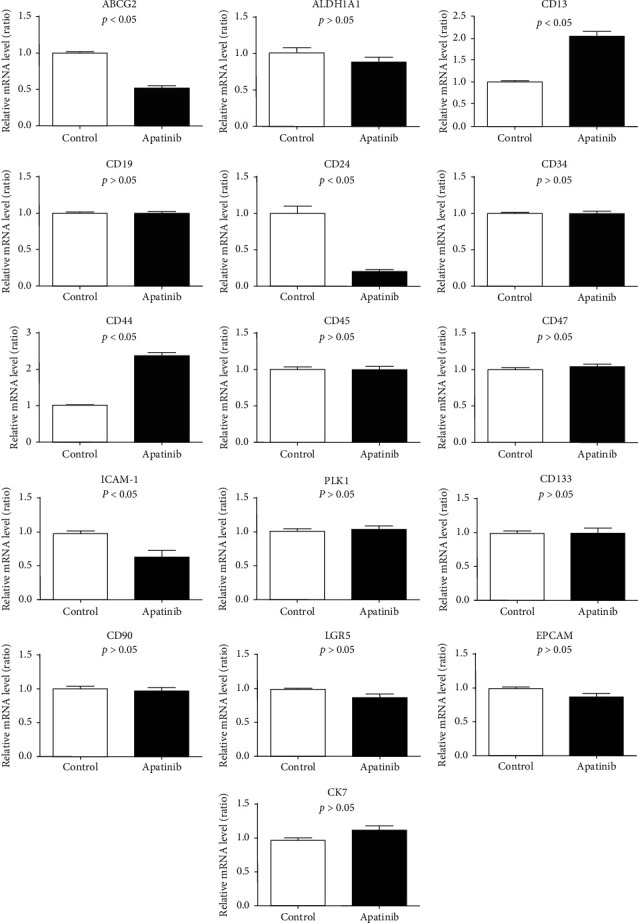
Effect of Apatinib on the surface markers of lung cancer stem cells.

According to the real-time PCR detection results, for the surface marker-related genes of lung cancer stem cells, the expressions of *CD13* and *CD44* were upregulated in the Apatinib group when compared to the control group, whereas the expressions of *ABCG2*, *CD24*, and *ICAM-1* were downregulated. There were no significant differences in the expressions of *DLK1*, *CD133*, *CD90*, *CD45*, *ALDH1*, *LGR5*, *CD34*, *CD47*, *EPCAM*, *CK7*, and *CK19* between both groups (Figure 2).

**Figure 3:**
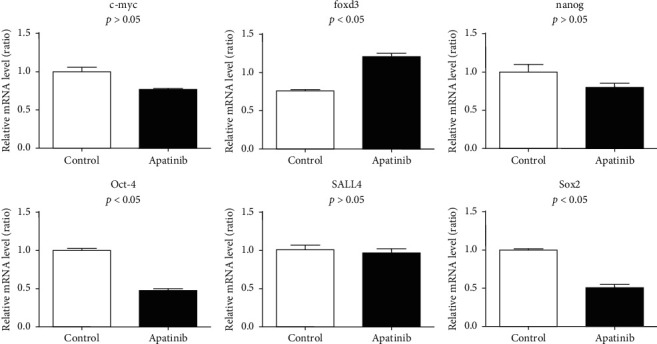
Effect of Apatinib on versatile regulators of lung cancer stem cells.

For the totipotent factor, *FOXD3* expression was elevated in the Apatinib-treated group, whereas *OCT4* and *SOX2* expressions were downregulated, and the expressions of *C-MYC*, *NANOG*, and *SALL4* showed no remarkable differences when compared to the control group (Figure 3).

**Figure 4:**
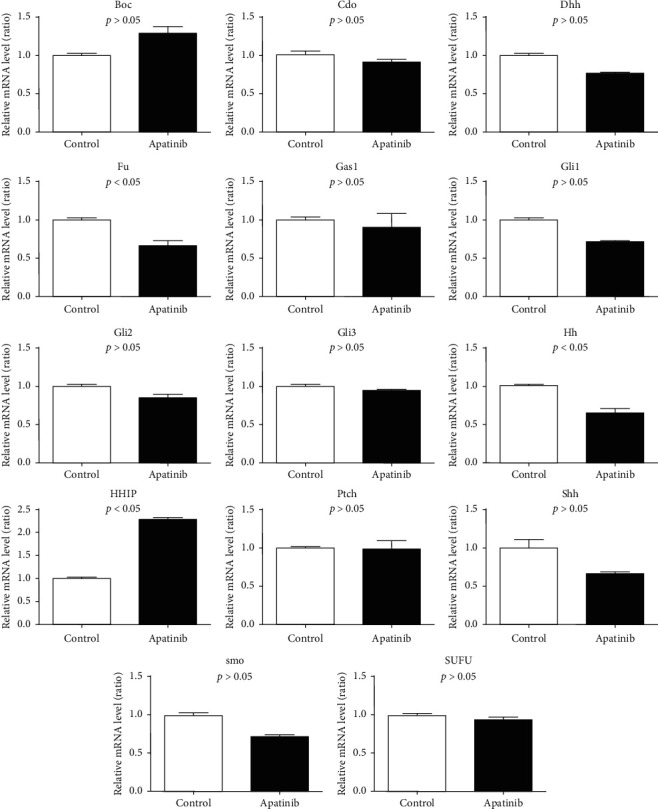
Effect of Apatinib on mRNA level of Hedgehog signaling pathway in lung cancer stem cells.

For the Hedgehog signaling pathway-related genes, the expression of *HHIP* apparently increased in the Apatinib treatment group, while the expressions of *HH*, *SHH*, and *FU* were downregulated, but there were no significant differences between *PTCH*, *SMO*, *GLI3*, *GLI1*, *GLI2*, *DHH*, *GAS1*, *CDO*, *BOC*, and *SUFU* expressions between both groups (Figure 4).

**Figure 5:**
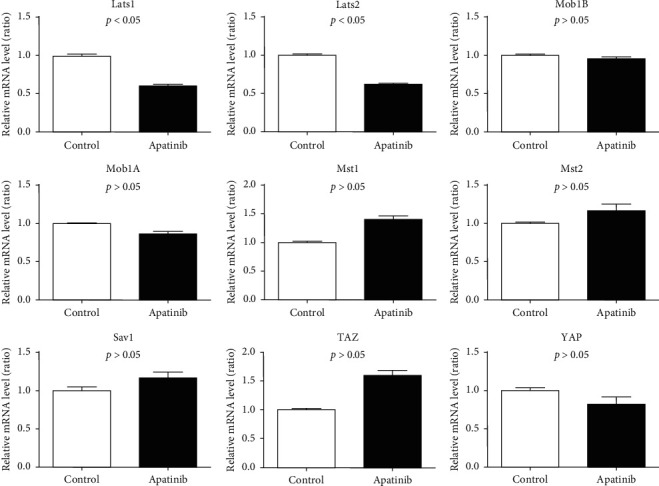
Effect of Apatinib on mRNA level of Hippo signaling pathway in lung cancer stem cells.

For the Hippo signaling pathway-related genes, the expressions of *LAST1* and *LAST2* were downregulated in the Apatinib-treated group, but there were no significant differences in the expressions of *MST2*, *MST1*, *SAV1*, *MOB1A*, *MOB1B*, *YAP*, and *TAZ* between the two groups (Figure 5).

**Figure 6:**
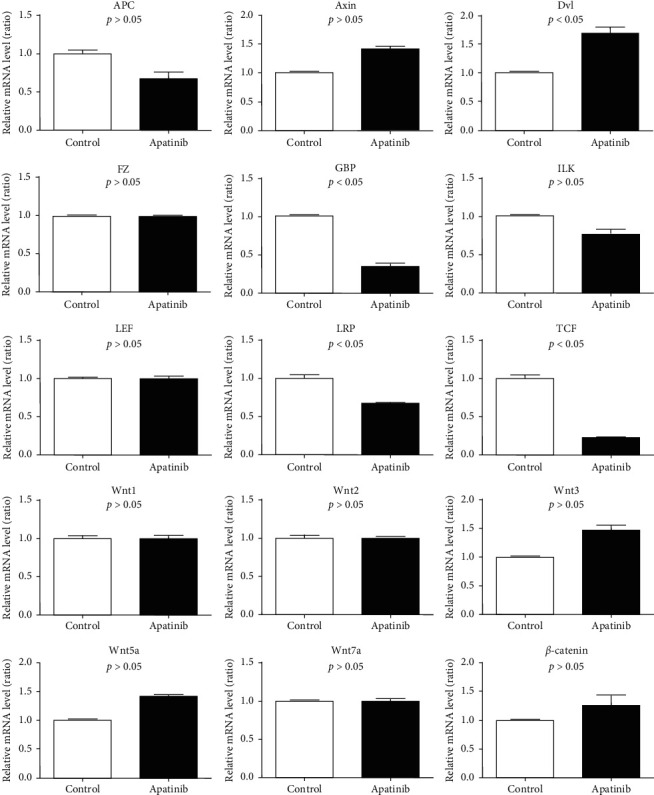
Effect of Apatinib on mRNA level of Wnt signaling pathway in lung cancer stem cells.

For the Wnt signaling pathway-related genes, the expressions of *GBP*, *TCF*, and *LRP6* were downregulated in the Apatinib-treated group, while the expression of *DVl* was upregulated, but there were no significant differences in the expressions of *APC*, *AXIN*, *FZ*, *β*-catenin (*CTNNB1*), *C-MYC*, *ILK*, *WNT*, *LEF*, and *CK1* between the two groups (Figure 6).

## 4. Discussion

Only a small fraction (<1.0%) of tumor cells in tumor tissues have tumorigenic properties of self-renewal, unlimited proliferation, and multiple differentiation potentials similar to those of normal stem cells. The formation and development of tumors are dominated by cancer stem cells (CSCs). A recent study analyzed gene expression profiles of several NSCLC primary cultures and cell lines and found that *NANOG*, *NOTCH3*, *CD44*, *CDKN1A*, *SNAI1*, and *ITGA6* were able to differentiate CSCs [10]. Lung cancer is one of the most common malignant tumors. With further studies carried out on lung cancer and LSCs, recent investigations have proven that TSCs also exist in lung cancer [11]. Several studies have shown that lung cancer stem cells (LCSCs) play an important role in the occurrence, development, recurrence, and metastasis of this malignant disease. Besides, research has shown that LCSCs are highly resistant to radiotherapy and chemotherapy. This high drug resistance rate of lung cancer could also be closely related to LCSCs, whose mechanism mainly includes the following: (1) tumor stem cells have a strong self-repair ability and low level of reactive oxygen species (ROS), which can avoid the damage of DNA sequence through a DNA repairing mechanism and ensure cell activity; (2) the drug is pumped out of the cell with the help of an ATP-binding cassette transporter, which decreases the intracellular drug concentration; (3) lung cancer stem cells are in a state of retention (stage G0) and cannot be effectively divided and proliferated [12]. As a result, certain drugs acting on the cell proliferation phase cannot produce an effect, resulting in drug resistance. In this study, we found that Apatinib could inhibit the expressions of stemness biomarkers *ABCG2*, *CD24*, *ICAM-1*, *OCT4*, and *SOX2* in lung cancer.

Further analyses showed that the Hedgehog signaling pathway is activated significantly in both NSCLC and small-cell lung cancer. The Hedgehog signaling pathway, which is involved in nicotine activation of lung cancer, is the first step in the activation of lung cancer [13]. In this study, Apatinib inhibited the Hedgehog signaling pathway and downregulated the *SHH*, *HH*, and *FU* of this signaling pathway. Therefore, Apatinib could be the first step to blocking the proliferation, invasion, and metastasis of NSCLC through the Hedgehog signaling pathway. The Hedgehog pathway is also involved in cancer resistance, such as cytotoxic chemotherapy, radiotherapy, and targeted therapy. Because the Hedgehog signaling pathway is related to ABC transporter, its inhibition is considered a chemosensitizer after chemotherapeutic drug resistance [14]. Della and other studies have shown that the Hedgehog signaling pathway is one of the mechanisms of drug resistance induced by *EGFR* inhibitors [15]. It could be suggested that Apatinib reverses chemoresistance by inhibiting the Hedgehog signaling pathway and, therefore, prolongs the PFS and OS of chemotherapy-resistant patients.

The activation of the Wnt signaling pathway is present in most lung cancer patients. Studies have shown that the Wnt signaling pathway has a significant effect on the occurrence, prognosis, and drug resistance of lung cancer [16, 17]. Our investigation revealed that Apatinib reduces the expressions of *TCF*, *GBP*, and *LRP6* in the Wnt signaling pathway significantly to inhibit the pathway, thus suppressing tumor and inhibiting the progression of lung cancer. The absence of any significant increase in the expressions of *APC*, *AXIN*, *WNT1*, *WNT2*, *WNT3*, *WNT5a*, *WNT7a*, and *β*-catenin in the Wnt signaling pathway within the Apatinib group could be due to the shorter drug-administration time; these target genes may be downregulated if treatment time is prolonged. Studies have demonstrated that the overexpression of *WNT1* gene in the Wnt signaling pathway can induce the apoptotic resistance of cancer cells [18]. The expression of the multiple drug resistance gene (*MDR1*) is also closely related to the activation of the Wnt signaling pathway [19]. Therefore, we reasonably speculated that the mechanism by which Apatinib can improve the prognosis of chemotherapy-resistant patients significantly might be through the inhibition of the Wnt signaling pathway.

The Hippo signaling pathway, like the Hedgehog and Wnt signaling pathways, is a key member of the stemness pathway of lung cancer. The main components of the Hippo signaling pathway include *MST1/2*, *LATS1/2*, and *YAP/TAZ*. *YAP* and *TAZ*, located on the human chromosome 11q22, are overexpressed in NSCLC and regarded as oncogenes of lung cancer [20]. Although *LATS1/2* was downregulated in our study, its upstream target, *MST1/2*, or downstream target, *YAP/TAZ*, did not increase or decrease. Therefore, we speculated that Apatinib could lessen the expression of oncogene *YAP/TAZ*, which in turn relieves the inhibition of the tumor suppressor genes *MST1/2* and *LATS1/2*; they may also play roles at the same time in the process Apatinib works. However, the specific mechanism of Apatinib in the Hippo signaling pathway still needs further research to confirm.

In summary, our investigation found that Apatinib inhibited the growth of NSCLC subcutaneous xenografts by inhibiting the Hedgehog, Hippo, and Wnt pathways, which suppressed the stemness nature of lung cancer cells and then inhibited the progression of lung cancer. The Wnt, Hedgehog, and Hippo signaling pathways are potential targets for the diagnosis and treatment of NSCLC. By inhibiting these pathways, not only could tumor proliferation, invasion, and metastasis be repressed, but it is also expected to be a significant target for reversing drug resistance in NSCLC. This study provides a theoretical basis for the use of Apatinib in advanced NSCLC patients with multidrug resistance. The limitation of our study is that it was carried out only at the animal level. In-depth research to verify and explore the molecular mechanism at the human level is required in the future.

## Figures and Tables

**Table 1:** Characteristics of the primers used for real-time PCR.

Genes	Primers (forward and reverse)	Products (bp)
PTCH	5′-TGGAACGAGGACAAAGCGG-3′	202
	5′-AGGCATAGGCGAGCATGAGTAA-3′	
SMO	5′-TCTCGGGCAAGACCTCCTACT-3′	146
	5′-CGCACGGTATCGGTAGTTCTT-3′	
GLI1	5′-CCAAGCACCAGAATCGGACC-3′	140
	5′-TTTGGTCACATGGGCGTCAG-3′	
GLI2	5′-AGGGATGACTGTAAGCAGGAGG-3′	152
	5′-CGGCACACAAACTCCTTCTTCT-3′	
GLI3	5′-GCACTAAGCGTTACACAGACCCA-3′	257
	5′-CTTCTCTGCCTTGACGGTTTTC-3′	
SHH	5′-CTGCTGGTATGCTCGGGACT-3′	106
	5′-CATTGGGGATAAACTGCTTGTAG-3′	
DHH	5′-GATGACCGAGCGTTGTAAGGA-3′	277
	5′-GTTATCAGCTTTGACCGACACG	
FU	5′-TTGCTGCCCAGTTGGTGTCA-3′	225
	5′-TGTGGTCGTATGGTCGCTCCT-3′	
GAS1	5′-ATCTGCGAGTCGGTCAAGGA-3′	133
	5′-TGCGCTGCTCGTCATCGTA-3′	
Cdon	5′-CTTTTCCAGCCGTCCAATAACT-3′	205
	5′-GTGCCACTGCTTTGAACCTTCT-3′	
BOC	5′-CATCACTGCCCTTAACAACCAC-3′	158
	5′-TGTTTCCCTCATCCACTTCAATC-3′	
HH	5′-TCACCGTCTGGCACCCTAGT-3′	120
	5′-CATGGCTCCTCTTGAACCCTG-3′	
HHIP	5′-GAGAAGGTGCCTGAATGGGAAC-3′	270
	5′-GGTGAGTGGAACAGGCTTTGA-3′	
SUFU	5′-GCCTTCGCTTCGCTCTTTC-3′	212
	5′-AGGCCGAAGCTGATGTAGTGC-3′	
MST1	5′-CGGGTCCCAGTAGCCAAGAT-3′	191
	5′-GTGTCATTACCCGTACCTTTGG-3′	
MST2	5′-ACCATCTGCCTTAGGAACGGA-3′	223
	5′-TAATTGCGACAACTTGACCGG-3′	
LATS1	5′-CCTATTAATGCCAGCATGAAACC-3′	220
	5′-CGTTGCTAGGGTGAGCTTGA-3′	
LATS	5′-GAGCAGATTGTGCGGGTCATT-3′	116
	5′-TGGTGGTAGGACGCAAACGA-3′	
SAV1	5′-GAATGCCACAGAATCAGGGGA-3′	291
	5′-GATGCCTGTATTGGGCCTTCTT-3′	
MOB1A	5′-AGTGGGAATCTGAGACAAGCTG-3′	224
	5′-GGTGCAGAACATTTGATTGGCT-3′	
MOB1B	5′-TGGGCAGATGGAACGAACA-3′	232
	5′-GAAGCTGGATCACAGGGTCAA-3′	
YAP	5′-GAGTTAGCCCTGCGTAGCCA-3′	272
	5′-GGCAGGGTGCTTTGGTTGATA-3′	
TAZ	5′-CCTGAAACTCCGCCACATCT-3′	235
	5′-CTGGTAGACGCCATCTCCTTTC-3′	
Notch1	5′-GTCAACGCCGTAGATGACC-3′	101
	5′-TTGTTAGCCCCGTTCTTCAG-3′	
Notch2	5′-ACTGTGAGGAGCAACTCGAT-3′	133
	5′-TCCACTTCATACTCACAGTTGA-3′	
Notch3	5′-TGACCGTACTGGCGAGACT-3′	67
	5′-CCGCTTGGCTGCATCAGCA-3′	
Notch4	5′-AACTCCTCCCCAGGAATCTG-3′	168
	5′-CCTCCATCCAGCAGAGGTT-3′	
JAG1	5′-GCCAGGAAGTTTCAGGGAGA-3′	264
	5′-GCTGGAGACTGGAAGACCGA-3′	
JAG2	5′-CTGACTGCCGCATCAACATC-3′	92
	5′-GCTACAGCGATACCCGTTGA-3′	
DLL1	5′-TCTCCTGATGACCTCGCAACA-3′	149
	5′-TCACACACGAAGCGGTAGGAG-3′	
DLL3	5′-CACTCAACAACCTAAGGACGCA-3′	212
	5′-CGAGGAAGGGTAGGGAAAAAG-3′	
DLL4	5′-GACCTCTCCACAGACACCTTTG-3′	299
	5′-TCCACTTCCAGCTCCTTCTTCT-3′	
HES1	5′-ATTCTGGAAATGACAGTGAAGCAC-3′	168
	5′-CACCTCGGTATTAACGCCCTC-3′	
HES5	5′-GAAAAACCGACTGCGGAAGC-3′	184
	5′-GACGAAGGCTTTGCTGTGCT-3′	
NGN	5′-CAGATTTGGTCCCATTTGTGAG-3′	279
	5′-ATGGCAACACTACATCCTGACC-3′	
Hey1	5′-GAAGCAGGTAATGGAGCAAGGA-3′	258
	5′-GAAGCGTAGTTGTTGAGATGCG-3′	
HEY2	5′-AAGGCTACTTTGACGCACACG-3′	146
	5′-GAGATGAGACACAAGCCGCAC-3′	
HES1	5′-ATTCTGGAAATGACAGTGAAGCAC-3′	168
	5′-CACCTCGGTATTAACGCCCTC-3′	
DV1	5′-AGAAGTCAGCTCTTGCCTCAGTT-3′	256
	5′-ATCTCATCAGTAGCACGACGAAG-3′	
AXIN	5′-TCTGGATACCTGCCGACCTTA-3′	271
	5′-TCTGCTGCTCGCTGTCGTT-3′	
APC	5′-CAAAACTGGAAACTGAGGCATCT-3′	225
	5′-ACTCTCCAGAACGGCTTGATACA-3′	
*β*-catenin	5′-GCCAAGTGGGTGGTATAGAGG-3′	192
	5′-GGGATGGTGGGTGTAAGAGC-3′	
GSK3B	5′-GTTAGCAGAGACAAGGACGGCA-3′	183
	5′-GCAATACTTTCTTGATGGCGAC-3′	
GBP	5′-AGGTGGCTCCTGACGCTAA-3′	180
	5′-CAGGCTGGAAGGGAAAGACA-3′	
TCF	5′-GAGTATGCCTACCTCAAAGCCA-3′	80
	5′-AGCCGCTTGATCTTCCCTG-3′	
FRZB	5′-ACGGAAACTGTAGAGGGGCA-3′	204
	5′-CAGTGTCCCGTGGAATGTTTAC-3′	
c-Myc	5′-TGCTGCCAAGAGGGTCAAGT-3′	160
	5′-GCTCCGTTTTAGCTCGTTCC-3′	
PKC	5′-GCTTATGCTGTCATGTCCCGG-3′	120
	5′-GATGATGAGGACTCCCCCCA-3′	
RHOA	5′-ATTGTTGGTGATGGAGCCTGTG-3′	83
	5′-GTGGGCACATACACCTCTGG-3′	
ILK	5′-GCAGTGAATGAACACGGGAAT-3′	78
	5′-CACCAGGTCCTCTGCCACTT-3′	
CD24	5′-GCTCCTACCCACGCAGATTT-3′	109
	5′-GGTGGCATTAGTTGGATTTGG-3′	
CD13	5′-CCGACATTGACAAGACTGAGCT-3′	175
	5′-ACCTTTCTGACATTGCCCTCC-3′	
CD44	5′-TCCAACACCTCCCAGTATGACA-3′	151
	5′-CTTTCTGGACATAGCGGGTG-3′	
CD90	5′-CGCTCTCCTGCTAACAGTCTTG-3′	213
	5′-GTTCGGGAGCGGTATGTGTG-3′	
CD45	5′-TGAGGAGCAAGGAAGCCAATC-3′	77
	5′-GCCACCAACTGAAGGCTGAAC-3′	
ALDH1	5′-TGCCGACTTGGACAATGCT-3′	277
	5′-GCCTCCTCCACATTCCAGTTT-3′	
ICAM1	5′-CCGTTGCCTAAAAAGGAGTTGC-3′	221
	5′-TGGCAGCGTAGGGTAAGGTTC-3′	
LGR5	5′-CGGGAAACGCTCTGACATACAT-3′	260
	5′-ACTTCTAAAAGCCTGGACGGG-3′	
CK7	5′-TGAAGAAGGATGTGGATGCTGC-3′	119
	5′-CAGCTCTGTCAACTCCGTCTCAT-3′	
CK19	5′-AGAATTGAACCGGGAGGTCG-3′	257
	5′-CCTGATTCTGCCGCTCACTA-3′	
CD34	5′-GCAACATCTCCCACTAAACCCTA-3′	156
	5′-GTCCTTCTTAAACTCCGCACAGC-3′	
CD47	5′-ACCTCCTTCGTCATTGCCATATT-3′	89
	5′-ATACACGCCGCAATACAGAGAC-3′	
DLK1	5′-CCCTTTGTGACCAGTGCGTG-3′	183
	5′-ATTCATAGAGGCCATCGTCCAG-3′	
EpCAM	5′-TAATCGTCAATGCCAGTGTACTTC-3′	101
	5′-AGCCATTCATTTCTGCCTTCAT-3′	
CD133	5′-TGGCATCTTCTATGGTTTTGTGG-3′	159
	5′-TCCTTGGTAGTGTTGTACTGGGC-3′	
OCT4	5′-GAGTGAGAGGCAACCTGGAGAAT-3′	292
	5′-ACCGAGGAGTACAGTGCAGTGAA-3′	
SOX2	5′-GGTTACCTCTTCCTCCCACTCC-3′	140
	5′-CGCTCTGGTAGTGCTGGGAC-3′	
NANOG	5′-GAGAAGAGTGTCGCAAAAAAGGA-3′	163
	5′-TGAGGTTCAGGATGTTGGAGAGT-3′	
FOXD3	5′-GCAACTACTGGACCCTGGAC-3′	142
	5′-TAAGCGCCGAAGCTCTGCAT-3′	
SALL4	5′-CATCAACTCGGAGGAGGACCA-3′	275
	5′-TGATGAGGACAGGTGGATTTTTAGT-3′	
*β*-actin	5′-AGTTGCGTTACACCCTTTCTTGAC-3′	171
	5′-GCTCGCTCCAACCGACTGC-3′	

## Data Availability

The data used to support the findings of this study are available from the corresponding author upon request.
